# 3D-Printed Liquid Cell Resonator with Piezoelectric Actuation for In-Line Density-Viscosity Measurements

**DOI:** 10.3390/s21227654

**Published:** 2021-11-18

**Authors:** Javier Toledo, Víctor Ruiz-Díez, Jaime Velasco, Jorge Hernando-García, José Luis Sánchez-Rojas

**Affiliations:** Microsystems, Actuators and Sensors Lab, Universidad de Castilla-La Mancha, 13071 Ciudad Real, Spain; victor.ruiz@uclm.es (V.R.-D.); Jaime.Velasco@alu.uclm.es (J.V.); jorge.hernando@uclm.es (J.H.-G.)

**Keywords:** 3D-printing, piezoelectric, PZT, actuators, oscillator circuit, low-cost, density, viscosity, monitoring, in-line

## Abstract

The in-line monitoring of liquid properties, such as density and viscosity, is a key process in many industrial areas such as agro-food, automotive or biotechnology, requiring real-time automation, low-cost and miniaturization, while maintaining a level of accuracy and resolution comparable to benchtop instruments. In this paper, 3D-printed cuboid-shaped liquid cells featuring a rectangular vibrating plate in one of the sides, actuated by PZT piezoelectric layers, were designed, fabricated and tested. The device was resonantly excited in the 3rd-order roof tile-shaped vibration mode of the plate and validated as a density-viscosity sensor. Furthermore, conditioning circuits were designed to adapt the impedance of the resonator and to cancel parasitic effects. This allowed us to implement a phase-locked loop-based oscillator circuit whose oscillation frequency and voltage amplitude could be calibrated against density and viscosity of the liquid flowing through the cell. To demonstrate the performance, the sensor was calibrated with a set of artificial model solutions of grape must, representing stages of a wine fermentation process. Our results demonstrate the high potential of the low-cost sensor to detect the decrease in sugar and the increase in ethanol concentrations during a grape must fermentation, with a resolution of 10 µg/mL and 3 µPa·s as upper limits for the density and viscosity, respectively.

## 1. Introduction

3D printing is a promising manufacturing alternative for microsystems that allows fast implementation and manufacturing of objects by successive layer-by-layer deposition from raw material with low-cost and high accuracy [1]. In this respect, various manufacturing processes and technologies have been introduced, and increased, over the years in a wide range of applications [2,3,4]. Currently, this technology can produce geometrically complex parts, electronic components and sensors [1,2,5,6,7,8] in a short period of time, compared to traditional manufacturing processes, which require high cost and time for customization.

The measurement of different physical and chemical parameters is essential in the engineering field. In this regard, different types of sensors have been fabricated. For example, strain sensors [9], pressure sensors [10], tactile sensors [11], accelerometers [12], optical sensors [13], biosensors [14,15], biomedical sensors [16], chemo sensors [17], and sensors for monitoring food quality [18] were fabricated and tested using various 3D printing technologies, such as fused deposition modelling (FDM), lamination and material jetting [19].

Focusing on one of the topics of this work, sensors based on resonators, works have also been reported. For example, in [20] a capacitive acoustic resonator was developed by combining three-dimensional (3D) printing and two-dimensional (2D) printed electronics techniques. In reference [21] a microwave cavity resonator was also presented for chemical sensing applications, where the resonator was comprised of a 3D split-ring resonator using polylactic acid (PLA) filament.

Nevertheless, only a few works regarding 3D-printed piezoelectric sensors have been published. For instance, in [22] the manufacturing routes of piezoelectric materials that have arbitrary piezoelectric coefficient tensors was introduced. In reference [23] an overview of 3D printing methods of piezoelectric materials using various poling techniques was described. In reference [24] a first characterization of the piezoelectric properties of 3D printed PVDF was presented. Finally, a low-cost micro-stereolithography technique with the ability to manufacture dense piezoelectric ceramic components was reported [25]. Despite the many technologies available, challenges still remain regarding the range of piezoelectric materials that can be successfully printed, since ceramics cannot be easily machined. In this way, 3D printing technology opens an effective pathway in geometrical flexibility. However, the piezoelectric properties limit the application of printed ceramics [26].

In this work, 3D-printed electromechanical resonant sensors actuated by piezoelectric films, to monitor liquid properties of aqueous solutions modeling fermenting grape must for application in the winemaking industry, will be demonstrated. Among the several advantages of using piezoelectric materials for the transduction of mechanical vibrations into electrical signals, we can highlight the wide frequency bandwidth, high sensitivity, high signal-to-noise ratio, high stability of the resonance frequency, low-size, light weight and low power consumption [27,28].

Several publications have already reported the use of piezoelectric resonators to monitor liquid properties [29,30,31,32,33,34] using the traditional manufacturing processes of silicon-based MEMS devices. One of the novelties of our approach is the implementation of a sensor binding 3D-printed liquid cells and commercial PZT (modified lead zirconate titanate PIC255 type 5A) plates with thin CuNi electrodes [35]. In our design, the liquid cell and the resonator are combined in one single and compact device, able to monitor in real-time the physical properties of a liquid once the appropriate signal conditioning circuit and calibration are carried out.

The implementation of our sensor presents as main advantages the low-cost and rapid manufacturing compared to the traditional fabrication processes of silicon-based MEMS devices [30,31,36,37,38]. Yet, the major benefit is related to the design of the liquid cell. In our case, the PZT actuators were attached outside the liquid cell, avoiding the possible deterioration of the actuators in aggressive media, and therefore contributing to a better performance of the sensor in various applications where reliability and sensor life span are critical.

In the following sections, we describe the design, fabrication and the characterization of the different sensors, and finally, the application as a real-time density-viscosity sensor.

## 2. Materials and Methods

### 2.1. Design and Optimization

One of the objectives was to design and fabricate a sensor combining 3D-printed liquid cells and commercial actuators featuring low resonance frequency of the vibration mode, recirculation of the fluid through the resonator structure, electrical wiring and PZT actuators without contact with the liquid, and all of these using a low-cost and fast-manufacturing process. To achieve these constraints different devices were considered and simulated to select a vibrational mode that allowed us to implement a simple and low-cost electronic circuit, based on a PLL oscillator [39] taking into account signal to noise ratio and frequency.

**Figure 1:**
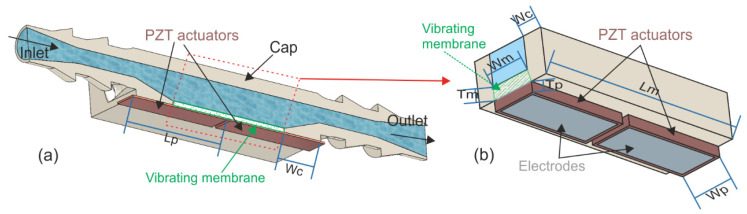
(**a**) Cross-sectional view of the cuboid 3D-printed liquid cell along a symmetry plane with the attached PZT actuators. (**b**) Schematic view with key dimensions defining the vibrating membrane (dimensions are not to scale).

Figure 1a shows a cross-sectional view of the design of the cuboid 3D-printed liquid cell along a symmetry plane with the attached PZT actuators, and Figure 1b shows a schematic view with key dimensions. The main feature of the cell was a thin membrane on one of the sides. The thickness of the 3D-printed membrane allowed for the actuation of the corresponding resonance modes, by the proper location and geometry of PZT piezoelectric patches. The frequency location of the resonance modes, affected by the liquid interaction, is a common procedure to study liquid rheological properties [29,39,40]. In this way, the integration of the membrane on the cell allowed for a compact easy-to-build sensor. In our case, the boundary condition of the vibrating membrane can be considered anchored on the four edges, as the rest of the 3D-printed cell, cap and walls were thick and rigid enough (see Table 1).

One of the advantages of our design was that there was no contact between the external PZT actuators and the liquid, avoiding dielectric losses through the wiring and protecting the actuators from the degradation induced by aggressive liquids. Furthermore, the resonating plate was loaded by the fluid onto one side only, reducing damping with respect to other designs with full immersion in liquid. The 3D printing approach also allowed for the easy implementation of two lateral connectors which, together with a peristaltic pump, allowed the recirculation of the fluid through the cell.

Our sensor was designed so that the rectangular membrane resonated with the out-of-plane, flexural 32-mode (numbers designating the nodal lines, including the anchors, along the long and short sides of the membrane). This low order mode was previously studied for liquid sensors and demonstrated an adequate trade-off between the conductance peak and the Q-factor in silicon-based MEMS devices [40,41]. In order to enhance the actuation of the 32-mode, consisting of two deformation lobes in opposite phase, two piezoelectric patches, driven in antiphase, were attached to the membrane, each covering one of the lobes [42,43]. As defined in Table 1 and Figure 1, the length of the PZT patches was extended to the edge of the cell to facilitate wiring, leading to a total length of Lp = Lm/2 + Wc.

In our work, the cell sensor was optimized and simulated with COMSOL [44], through a modal analysis, considering the dimensions of the actuators and liquid cell. This software package has been tested before with PZT interfaces anchored to structural materials demonstrating good performance for modal analysis, in comparison with the experiment [45]. In addition, COMSOL is a well-established FEM suite for fluid-structure interaction problems in the frequency domain [46].

In our case, the modal analysis was applied mainly to validate the nature of the modal shape (32-mode). The cell, filled with water and with the externally attached PZT layers, was discretized by 11,024 3D elastic solid second order (or quadratic) elements. The meshing featured 1536 elements for the PZT patches and 12,560 elements for the 3D printed body. The inner fluid was discretized with 8432 3D fluid elements. These values were determined by a trade-off between accuracy and computing time. A mesh convergence study determined that a higher resolution did not affect the modal frequencies more than 100 Hz around the approximately 19 kHz of the vibration mode of interest. Numerical calculations included both, the structural with piezoelectric interfaces on one side, and structural with thermoviscous acoustics interface for the liquid. Table 2 shows the mechanical properties of the different components employed in the simulation process.

**Figure 2:**
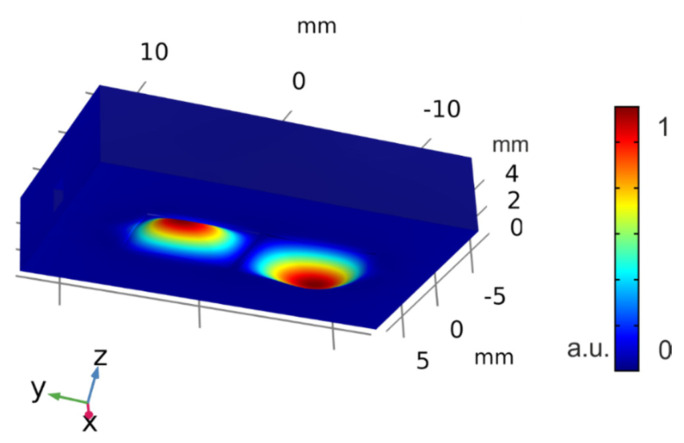
Simulation results from the modal analysis showing the 32−mode shape in the piezoelectric actuated side of the cell. The color scale represents the amplitude of deformation in arbitrary units.

As it can be observed in Figure 2, the modal analysis confirmed the presence of the out-of-plane 32-mode near 19 kHz with water inside the cell. As it is described in Section 2.3, the resonance parameters and the modal shape were determined by optical measurements under immersion in water, demonstrating that the optimized vibration mode corresponded to the 32-mode.

### 2.2. Fabrication

Once the design of the sensor was determined, different 3D-printed liquid cells were analyzed considering the material, the fabrication parameters, and the different post-treatment processes of the surface. Regarding the 3D printing materials for the liquid cell, the most widely used were analyzed. Some of them such as polylactic acid (PLA) and acrylonitrile butadiene styrene (ABS) [47], were discarded due to their low chemical resistance or modulus of elasticity, which are critical parameters for the final application as a density-viscosity sensor [48]. Other materials such as carbon fiber reinforced polyamide (PA-CF) [49], polyethylene terephthalate (PET), and silicone-based resins with different modulus of elasticity, were selected.

The fabrication parameters were adjusted to maximize the dimensional precision in the membrane thickness while assuring a maximum density, yet compact cell solid. Since additive manufacturing is based on a layer-by-layer deposition, the layer thickness is one of the main important parameters: the lower the layer height, the lower the dimensional tolerances in the membrane thickness, and the greater the solid compactness. In addition, smoother surfaces could be obtained by a lower layer thickness. Another important parameter was the temperature in order to improve the adhesion between the layers. Some of the key parameters of the fabrication of the 3D-printed cell are summarized in Table 3.

The PA-CF and PET cells were manufactured with a fused deposition modeling (FDM) printer [50], while the resin cells were produced with two stereolithography (SLA) printers [51,52]. As described in Table 3, the SLA printers offer the highest resolution, and specifically, the Rigid10k resin offers the highest modulus of elasticity (E) and, as indicated in their datasheet [53], an excellent chemical resistance (less than 0.1% weight change over 24 h in water and various solvents). These characteristics resulted in the outstanding behavior of liquid cell observed in the final application.

**Table 3:** Fabrication parameters and material properties used for the 3D-printed liquid cell. RT = room temperature.

Material	Printer	E [GPa]	Temperature [°C]	Printer Resolution	UV Curing
PA-CF [54]	FDM [50]	8.38	250	Z: 120	-
PET [55]	FDM [50]	1.93	240	Z: 120	-
Black resin [56]	SLA printer 1 [51]	1.75	RT	XY: 30 Z: 20	10 min
Rigid 10K resin [57]	SLA printer 2 [52]	10	RT	XY: 25 Z: 50	1 h, 70 °C

**Figure 3:**
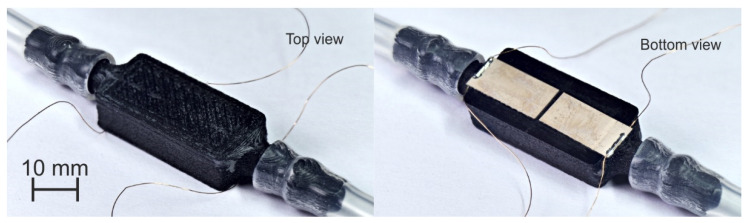
Top and bottom view of the PA-CF fabricated liquid cell. (Wc = 2.5 mm, Tm = 0.5 mm and Lm = 16 mm).

Regarding the attachment of the PZT actuators, a manual scribing and cut procedure was performed out of the commercial PZT plates [35]. Then, the patches were glued to the cell with an instant adhesive [58] being separated 1 mm from each other along their length, what allowed us the wiring and soldering of the electrical contacts outside the liquid cell, and prevented from any possible short-circuit between both patches (see Figure 3).

Once the fabrication parameters and materials were defined, different devices were fabricated and tested. For example, Figure 3 shows the initial fabricated cell sensor with PA-CF. In this case, the width of the liquid cell wall (Wc) is 2.5 mm, the thickness (Tm), width (Wm) and length (Lm) of the vibrating membrane are 0.5, 5 and 16 mm, respectively, as shown in Table 1.

**Figure 4:**
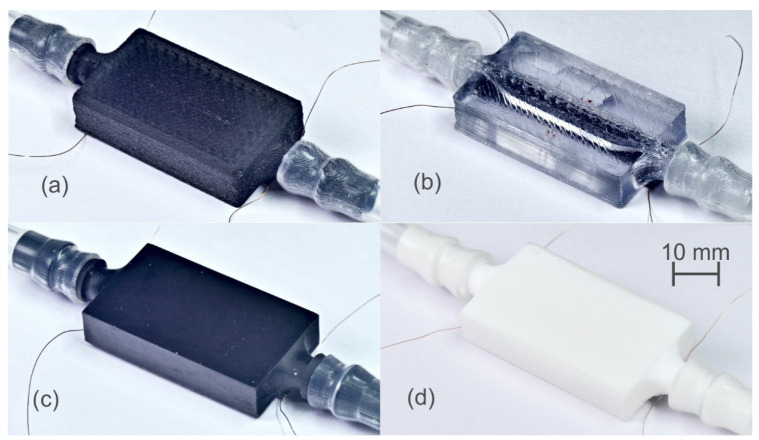
Top view of the fabricated liquid cells using different materials. (**a**) PA-CF, (**b**) PET, (**c**) black resin and (**d**) Rigid10K resin.

Similar structures were fabricated with other four different materials, increasing the width of the cell wall to 5 mm, as this was shown to improve the anchoring of the membrane and resonance peaks. Figure 4 shows a top view of the fabricated structures with the same dimensions.

**Figure 5:**
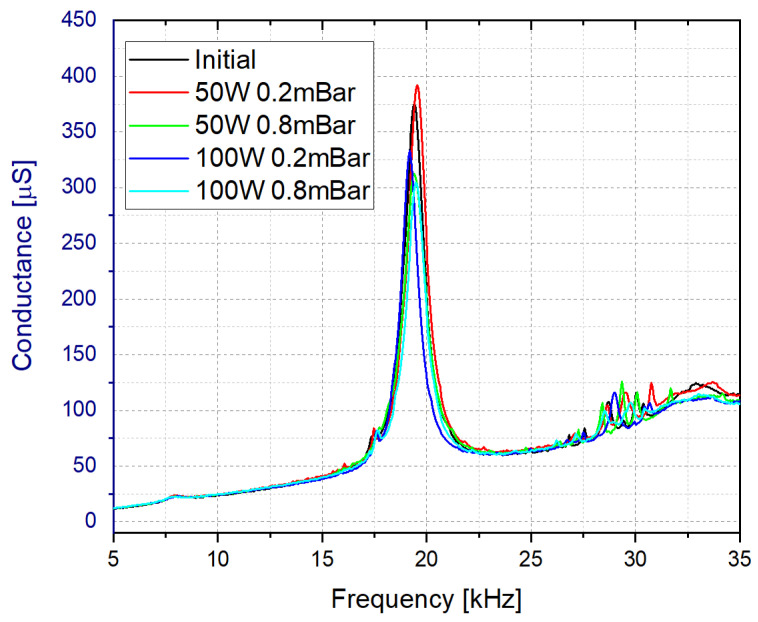
Effect of the plasma post-treatment in the frequency response of the sensor when immersed in water.

As shown below, the structure fabricated with the Rigid10K resin demonstrated the best performance in terms of Q-factor and conductance peak. Nevertheless, as it was observed during the initial measurements, a post-treatment process was required due to the low wettability of the resins [59,60] associated with generation of bubbles affecting the stability of the measurements. Different techniques were tested. Firstly, ultrasonic baths with inorganic solvents and acids, with different concentration and temperature conditions were tried. However, the results were not successful due to poor and difficult infiltration of the chemicals in the inner part of the liquid cell and the chemical resistance of the cells. For this reason, a post-treatment with oxygen plasma was selected as the best option. This process was already proven to be effective in improving the wettability of polymers [60]. In our case, the tests were conducted in a vacuum chamber equipped with a radio frequency plasma generator using different conditions in terms of plasma power, oxygen pressure and process time. Several experiments were conducted and eventually, a plasma treatment for 5 min with 50 W and a base pressure of 0.2 mBar of O_2_ were proved to be adequate to improve the wettability of the surface. As illustrated in Figure 5, it was checked that the plasma treatment maintained the resonance frequency response roughly constant.

**Figure 6:**
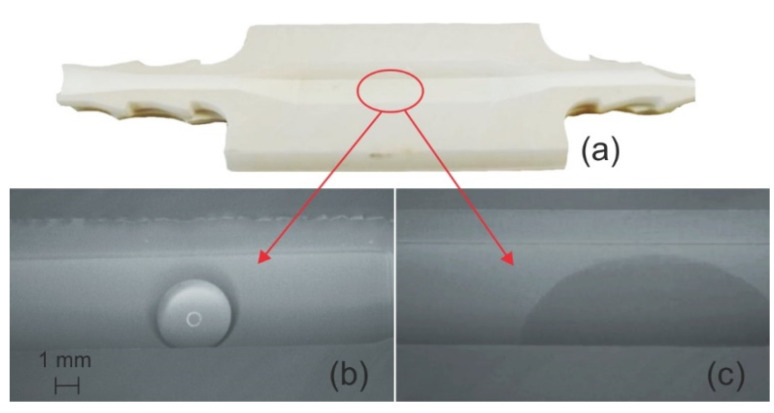
(**a**) Cross-sectional picture of the liquid cell fabricated with Rigid10K resin. Drop water on the inner surface (**b**) before and (**c**) after the post-treatment in a plasma chamber with O_2_.

The effectiveness of the plasma post-treatment was also verified by breaking a liquid cell into two parts along the axis and observing the behavior of a drop of water on the inner surface of the cell fabricated with the Rigid10K resin. As it can be observed in Figure 6c, the plasma treatment improved the wettability of this resin producing the spreading of the drop water across the surface. This step was crucial to prevent the generation of bubbles and make the measurements more stable with the liquid flowing through the cell.

### 2.3. Characterization

Once the proposed design and the key parameters of the fabrication process were discussed, the device structures were characterized considering various printing materials and slight variations in the geometry of the cell, namely the width of the cell wall (Wc), and the thickness (Tm) and the length (Lm) of the vibrating membrane. In our case, this comparison was performed with the help of the frequency spectrum of the sensor, obtained by means of an impedance analyzer. The membrane width, Wm = 5 mm, was maintained in all the structures.

To check the effect of the cell wall width (Wc), only one of the materials, PA-CF, was considered. This material required some harsh printing conditions, such as the high printing temperature or the larger diameter of the impression nozzle. Therefore, if a cell fabricated with PA-CF does not leak, under certain wall conditions, the rest of the materials are not expected to leak either. In this case, the cell was fabricated with different wall widths, being the rest of dimension as in Table 1. Our results showed that the structure fabricated with Wc = 5 mm presented the best performance in terms of the Q-factor and the conductance peak for the 32-mode in water.

**Figure 7:**
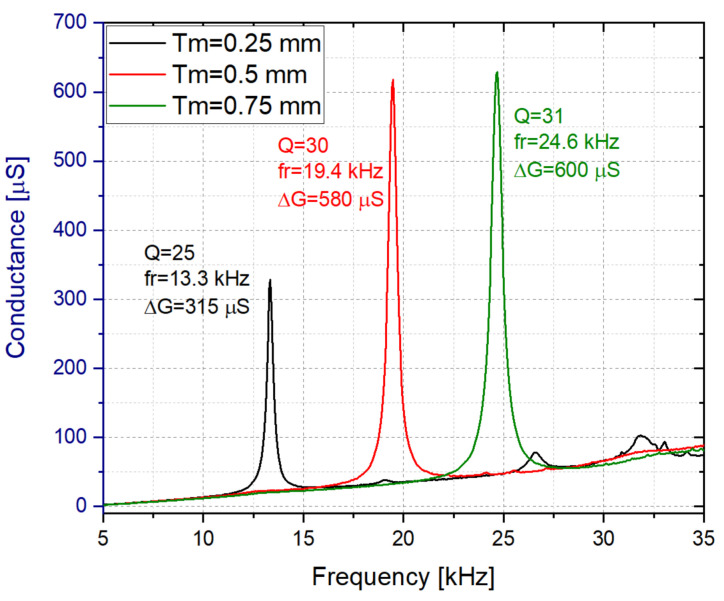
Conductance spectrum in water for the Rigid 10K resin based liquid cell with Tm = 0.25, 0.5 and 0.75 mm (Wm = 5 mm and Lm = 16 mm).

The effect of the length and the thickness of the membrane on the impedance response were also studied. As illustrated in Figure 7, in this case for the Rigid 10K resin with water inside, the membranes with Tm = 0.5 and 0.75 mm presented similar Q-factors and conductance peaks, despite the fact that a higher thickness is expected to produce a higher Q-factor [61]. Since a lower frequency allows for simpler electronic designs, the vibrating membrane with Tm = 0.5 mm was selected.

**Figure 8:**
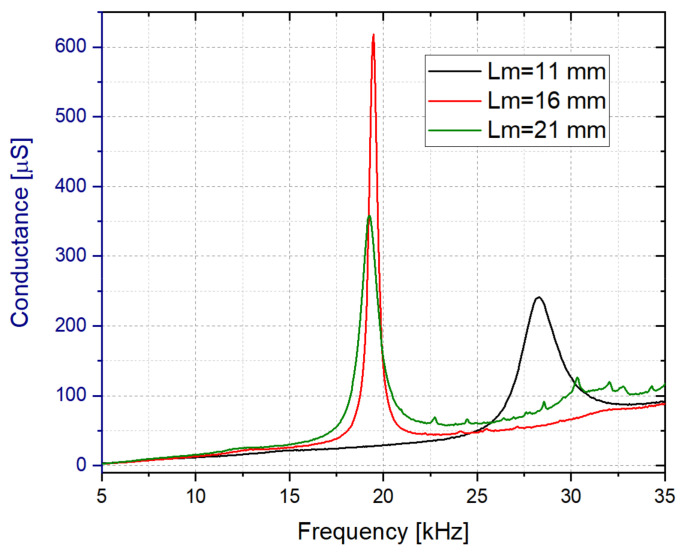
Conductance spectrum in water for the Rigid 10K resin based liquid cell with Lm = 11, 16 and 21 mm (Wm = 5 mm and Tm = 0.5 mm).

In the next step, the length of the vibrating membrane, Lm, was also studied. In this case, the structure with Lm = 16 mm presented the highest Q-factor and conductivity for the out-of-plane 32-mode (see Figure 8).

**Figure 9:**
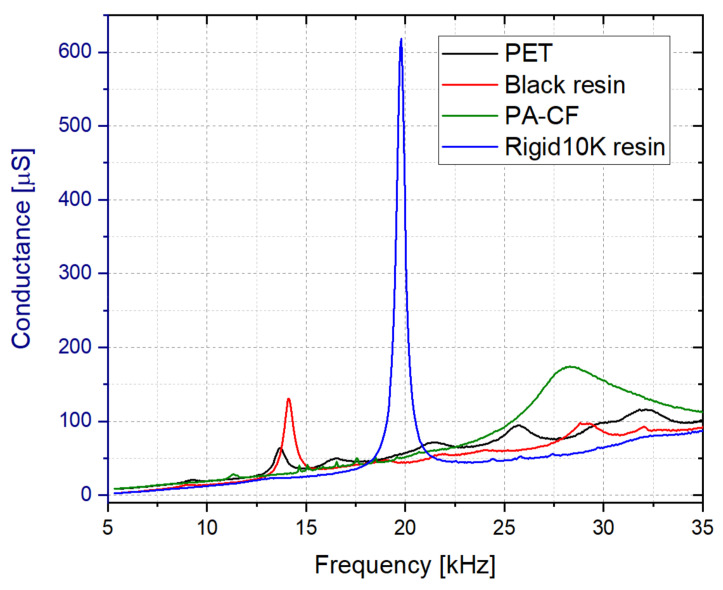
Conductance spectrum measured in water for the cells fabricated with different materials (Lm = 16 mm, Tm = 0.5 mm and Wc = 5 mm).

As summary, after evaluating all the previous conductance spectrums, it was concluded that the optimal dimensions corresponded to a 5 mm wide liquid cell wall surrounding a 0.5 mm thick and 16 mm long vibrating membrane. In a final comparison, and using the optimal dimensions just mentioned, the impedance response of the cells fabricated with the different materials of Table 3 were obtained. As it can be seen in Figure 9, the best performance corresponded to the sensor fabricated with the Rigid10K resin, since it presented the best Q-factor and conductance peak at a low resonance frequency. This conclusion was confirmed by the serial fabrication of seven sensors using the Rigid10K resin and obtaining a resonance frequency of 19.4 ± 2 kHz and a Q-factor of 30 ± 2 in water.

**Figure 10:**
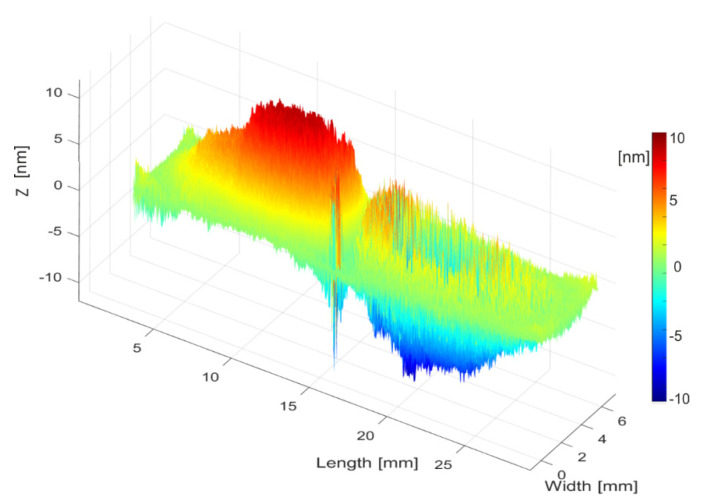
Picture of the optically measured 3D vibration of the detected resonance at 19.4 kHz in the fabricated cell. The modal shape corresponds to the out-of-plane 32-mode. The color scale represents the out-of-plane deformation when the cell patches were excited at 5 V.

In addition, it was also checked that the electrically detected resonance at 19.4 kHz corresponded to the out-of-plane 32-mode. For this analysis, we used an optical tool from Optonor (MEMSMap 510 [62]), which consisted of a speckle pattern-based interferometer capable of building a map of both the out-of-plane and the in-plane motion of the device surface. Figure 10 confirmed that the experimentally detected modal shape matched the out-of-plane 32-mode previously obtained in the simulation process (see Figure 2).

## 3. Application and Results

Once the designed cell resonator was fabricated and characterized, we focused on the final application as a density-viscosity sensor, and the results obtained in aqueous solutions. In this section, the oscillator circuit, the calibration process and finally the measurements performed in real-time and in-line for the monitoring of a set of artificial model solutions of grape must, representing the stages of a wine fermentation process, are described.

### 3.1. Phase-Locked Loop Based Oscillator Circuit

**Figure 11:**
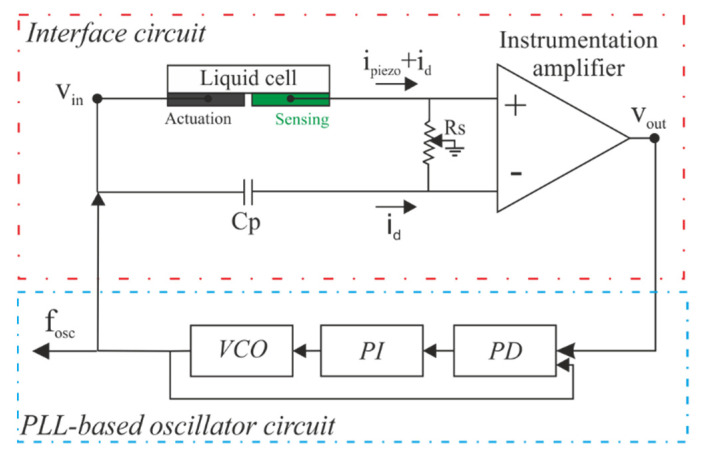
Schematics of the PLL-based oscillator and the interface circuit.

The simultaneous determination of density and viscosity of liquids, through measurements of the resonant frequency and the quality factor of a mechanical resonator, is challenging due to the low Q-factors and parasitic effects present in liquid media [63]. For this reason, an interface circuit, based on two PZT patches in a two-port configuration, one patch for actuation and the other for sensing, was implemented (see Figure 11). Nevertheless, our results showed that a dielectric current (i_d_) across the ports has a significant contribution to the output current. To reduce this effect, a capacitance (Cp) of approximately 10 nF was introduced in the inverting input of the instrumentation amplifier to subtract the dielectric current from the piezoelectric PZT actuator [64].

**Figure 12:**
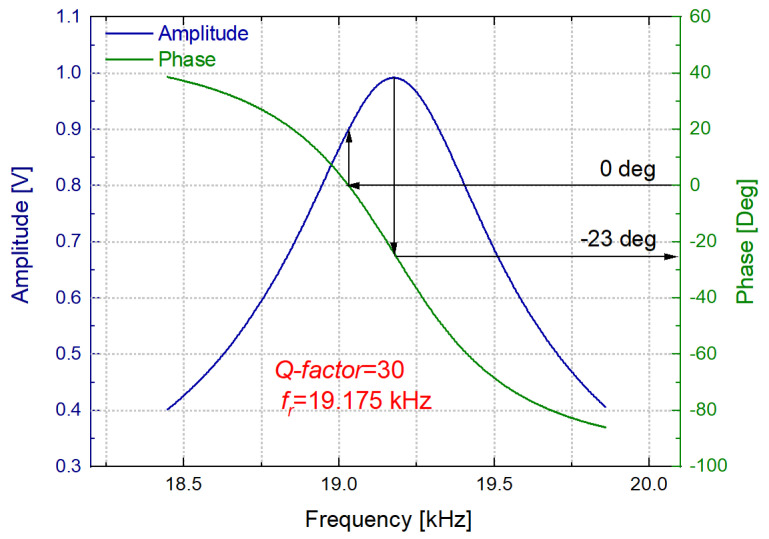
Open loop response of the interface circuit for the 32−mode in the grape must model solution N_1_.

Due to this procedure, a clear resonance with a remarkable phase transition was obtained in an open-loop measurement of the interface circuit with the liquid called N_1_ (water and sugars as defined below) inside the cell (see Figure 12). However, a phase shift of about −23° was found at the resonance frequency. To compensate for this phase shift, the resonator and interface circuit were finally connected as the feedback loop of an oscillator based on a Phase-Locked Loop (PLL) instrument.

The working principle of this instrument, called demodulation or phase-sensitive detection, rests on mixing the measured signal with a reference frequency followed by low-pass filtering. In our case, the PLL instrument used (Zurich HF2LI [65]) could track the oscillation frequency (f_osc_) and the gain of the interface circuit (G_osc_ = V_out_/V_in_) with the help of three blocks: a software-based voltage-controlled oscillator (VCO), a proportional-integral controller (PI) and a phase detector (PD). The parameters of the VCO to be configured are delay, gain and bandwidth. The gain parameter specifies the ratio of frequency shift on the VCO output to voltage change on the input. The correct choice of the bandwidth affects the signal-to-noise ratio (SNR), being the value selected in our case of 10 Hz. The phase detector is a software-controlled block which allows to choose the appropriate settings according to the frequency, phase and properties of the reference signal from the VCO. The output of the PD block is the input signal of the PI where the parameters can also be set via software. The schematic of all the blocks is presented in Figure 11.

### 3.2. Calibration Procedure

Once the parameters of the resonant structure could be acquired with the previously described PLL-based oscillator circuit, a calibration process was required in order to determine the density-viscosity of the liquid which circulated through the 3D-printed liquid cell. This required a two steps calibration process, each with adjustable parameters. In the first step the resonance parameters, f_r_ and Q-factor, were obtained, and in the second step the density and viscosity were estimated through the process described below.

It is worth pointing out that the range of viscosities that the resonator can measure is limited by the in-liquid Q-factor. Viscosities as high as 500 mPa·s have been measured with piezoelectric MEMS devices [39]. In the case of 3D-printed cells, the relatively lower resonance peaks restrict the type of applications. Nevertheless, the results presented in this section demonstrate the performance of the density-viscosity sensor for the in-line monitoring of aqueous solutions that model a fermenting grape must, relevant to the winemaking industry.

#### 3.2.1. Preparation of the Model Solutions of Grape Must

In order to confirm that the sensor is valid for the in-line monitoring of a real grape must fermentation, a set of model solutions were prepared for test purposes. The model solutions N_1_–N_9_ represented different stages of the corresponding fermentation process according to their mixture of glucose, fructose, ethanol and glycerol dissolved in water (see Table 4) [66]. As it can be noted, a decrease in fructose and glucose concentration from 110 to 2 g/L and 100 to 1 g/L, respectively, took place when changing from N_1_ to N_9_. Similarly, the glycerol and ethanol concentration increased from 0 to 9 g/L and up to 14% *v*/*v*. These model solutions were analyzed with a commercial benchtop density-viscosity meter (Anton Paar DMA4100M) and compared with the measurements from the fabricated cell resonator. 

#### 3.2.2. Calibration Process

As mentioned, a two-step calibration process was necessary to determine the density and viscosity of the liquid. In the first step of the calibration process, the oscillation frequency, f_osc,_ and the gain amplifier, G_osc,_ were transformed into the resonance parameters, namely the resonant frequency (f_r_) and Q-factor. Thanks to the phase shift compensation provided by the PLL, f_r_ was obtained as the oscillation frequency for each liquid. On the other hand, the Q-factor was calculated from the circuit output gain, G_osc_ [39].

**Figure 13:**
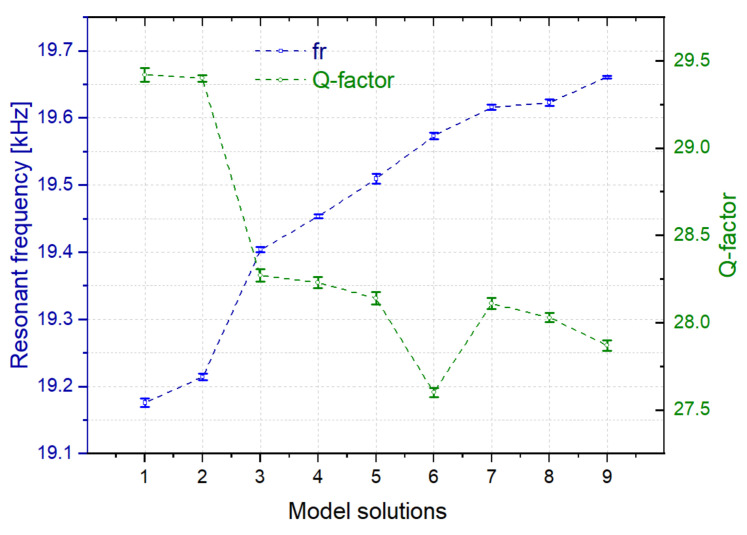
Mean values and deviations of the resonant frequencies and Q-factors of the sensor immersed in different model solutions of grape must at 20 °C.

Figure 13 shows the mean values and deviations of the resonance parameters of a Rigid 10K resin-based sensor for the model solutions N_1_ to N_9_, in static conditions (no fluid flow), obtained after five open-loop measurements of the interface circuit. The deviation error values were below 5 Hz for f_r_ and 0.04 for Q-factor in the worst case. In this case, the significant changes in the composition of the model solutions were sensed through the resonance parameters, proving the capability of the sensor to monitor the evolution of the process.

It must also be considered that due to the influence of the slight temperature changes and the electronic noise from the circuit and the measurement setup, random fluctuations in the measured variables were present. In our case, oscillator circuit output noise is present in both, amplitude and phase noise. The former as well as the gain of the interface circuit (G_osc_) are proportional to the Q-factor, leading to the output voltage noise proportional to the variance of the Q-factor. The phase noise affects the oscillation frequency (f_osc_), as given by the Allan variance, and according to the reference [67] is higher for lower Q-factors. In this way, random fluctuation of f_r_ was obtained directly through the Allan deviation of f_osc_ [68], and the uncertainty in Q-factor was obtained by error propagation from G_osc_ [40]. These fluctuations determine the sensor resolution obtaining an Allan deviation below 7 mHz and a standard deviation below 30 mHz for f_r_, and a deviation of 0.015 for Q-factor in the worst case. Therefore, with a Q-factor deviation of 0.015 over the value of 30, the estimated signal-to-noise ratio was 66 dB.

In the second step of the procedure, the density-viscosity values were obtained from the previous measurements of f_r_ and Q-factor calibrated against the density-viscosity values obtained with the density-viscosity meter mentioned before. The process was performed as follows. These resonance parameters are related to the mechanical properties of the resonators, i.e., resonator equivalent mass (m) and natural frequency in vacuum (f_o,vac_), and the fluid conditions, i.e., distributed damping associated with the liquid (g_1_) and the distributed equivalent added mass (g_2_), both per unit length (L). These variables can be separately determined as defined in Equations (1) and (2) [38,69,70]. Nevertheless, the dependence of g_1_ and g_2_ on the viscosity (*µ*) and density (*ρ*) of the fluid is only available for simple geometries and particular cases [71]. For this reason, a Taylor series expansion of g_1_ and g_2_ in terms of the density and viscosity was applied as in Equations (3) and (4) [72,73]. The equations were solved iteratively to obtain the values of the coefficients C_1_, C_2_, C_3_ and C_4_ that fit best to the experimental data for the model solutions.

(1) $$f_{r}=\frac{f_{o,vac}\sqrt{1-\frac{1}{2Q^{2}}}}{\sqrt{1+\frac{Lg_{2}}{m}}}$$

(2) $$Q=\frac{2\pi \sqrt{1+\frac{Lg_{2}}{m}}}{\frac{Lg_{1}}{m}}f_{o,vac}$$

(3) $$g_{1}=C_{1}\sqrt{f_{r}}\sqrt{\rho \mu }+C_{2}\;\mu$$

(4) $$g_{2}=C_{3}\rho +\frac{C_{4}}{\sqrt{f_{r}}}\sqrt{\rho \mu }$$

**Figure 14:**
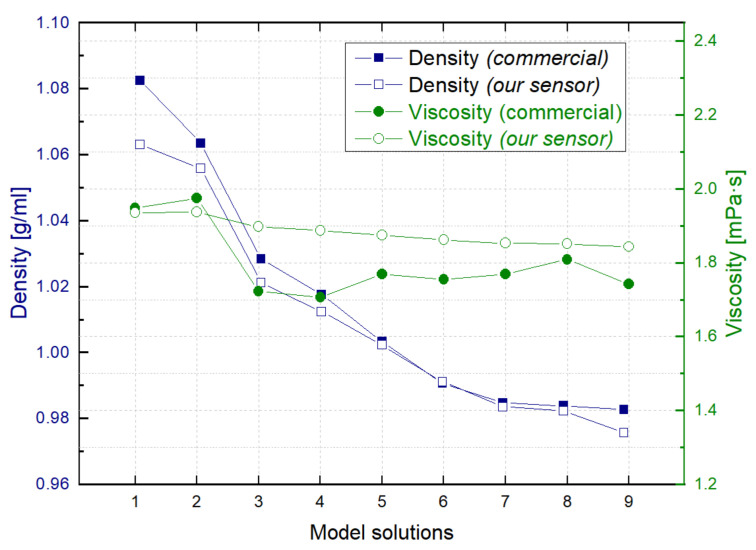
Density and viscosity values measured with the commercial density-viscosity meter and with our sensor cell for the model solutions.

Figure 14 shows the results of the estimated density and viscosity values using our sensor and the density-viscosity meter at 20 °C where an almost linear decrease in the density can be observed, as the fermentation progresses. In this case, the measurements were carried out with the model solutions in static conditions and verifying that the resonator parameters were maintained after each measurement and cleaning cycle with water flowing through.

Once the density and viscosity for each liquid were known, both the error associated with the calibration process and the resolution related to the random fluctuations could be determined. The error terms were calculated as the deviation between the density-viscosity values estimated from our sensor and the values measured with the commercial instrument, obtaining a mean error term of 5.35% for the viscosity and 0.54% for the density.

The density-viscosity resolutions were translated from the previously measured random fluctuations of 7 mHz for the resonant frequency and 0.015 for Q-factor [40]. In this case, the values obtained for all the model solutions were below 10 µg/mL and 3 µPa·s, for density and viscosity, in the worst case. These resolutions were comparable for all model solutions due to the similarity of the density and viscosity values of each model solution. The resolution and the calibration error obtained were better for the density than for the viscosity. This occurred because the Q-factor, determined from the measurement of G_osc_ in the interface circuit, presented a worse resolution than the frequency measurement.

Regarding the sensitivity of the sensor, the values were obtained from the derivatives of the resonant frequency and Q-factor with respect to the density and the viscosity. In this case, the sensitivity values of the resonant frequency were 1815 Hz/mPa·s and 4857 Hz/g/mL, and for the Q-factor the sensitivity values were 7.3 1/mPa·s and 19.7 1/g/mL. As these values indicated, the higher sensitivity corresponded to the resonant frequency with respect to the density. Therefore, in the next in-line and real-time monitoring procedures, only the resonant frequency is monitored.

### 3.3. Real-Time and In-Line Monitoring of Density and Viscosity

In the previous section, the potential of our sensor to monitor the density and viscosity of different model solutions of grape must was demonstrated. Nevertheless, one of the main objectives of the proposed design is the in-line monitoring of a liquid flowing through the cell. For this reason, we applied the same procedure, that was developed for the model solutions, to different real-time and in-line measurements. In this case, we focused only on the resonance frequency since, as it was previously demonstrated, is one of the key parameters to monitor a real fermentation process.

**Figure 15:**
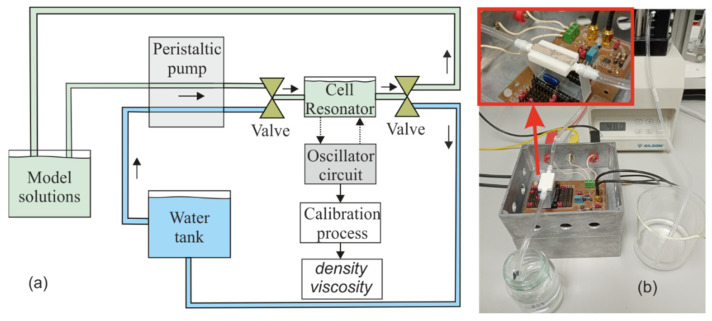
(**a**) Schematic and (**b**) photograph of the setup for the real-time monitoring of model solutions of a grape must fermentation process. Piezoelectric actuators are shown in the inset to (**b**).

In Figure 15, a schematic and a photograph of the setup for the monitoring of the model solutions is presented. In our setup, a peristaltic pump, Minipuls 3 from Gilson [74], was selected due to the high-performance, low-pulse and the possibility of use interchangeable pump heads for delivering a diverse variety of liquids without altering the critical components within the liquid. This pump injected the model solutions into 3D-printed liquid cell with a flow rate of 10 mL/min. In this case, the connexion between the liquid cell and the peristaltic pump was completed with a silicone tube with an inner diameter of 3.17 mm and output diameter of 4.87 mm which has chemical resistance to the liquids employed [75]. In addition, the valves allowed the recirculation of water to clean the interior of the liquid cell. As previously commented, the PZT actuators and wire connections to the electrodes were located at the bottom of the structure.

#### 3.3.1. Real-Time Monitoring

In the first set of measurements, identified as real-time, the systematic procedure was as follows. First, the model solutions were injected into the flow cell with the peristaltic pump, while the measurement of the resonance frequency was carried out following the same procedure as in the previous sections. After that, the valves were switched to allow water injection into the cell, cleaning the resonator and removing any remnants of the previous liquid. To check that the cleaning process was successful and effective, the value of f_r_ was registered before and after each measurement in water, obtaining a value of 19,420 ± 10 Hz.

**Figure 16:**
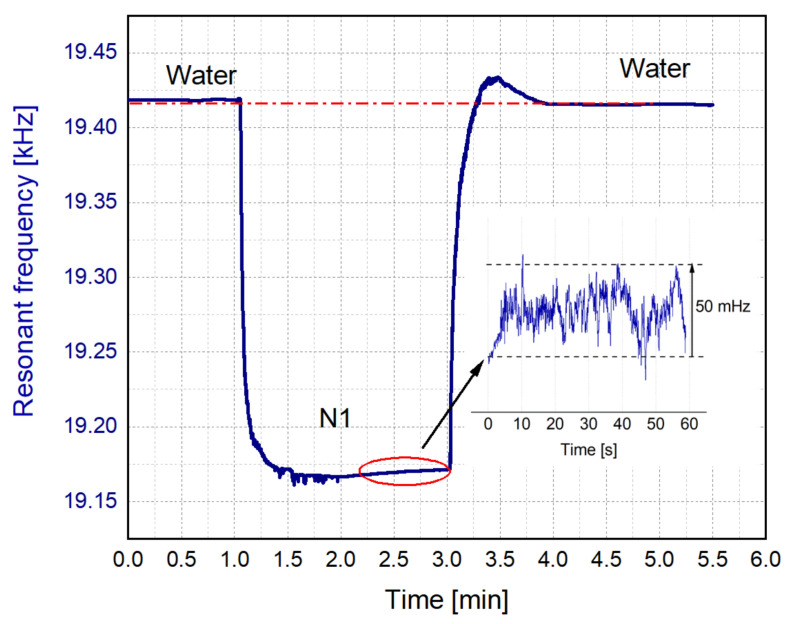
Real-time measurement of the resonance frequency while the sequence of water-N_1_-water liquids circulated through the liquid cell. Inset: Frequency stability for the model solution N_1_ after a recirculation time of one minute.

Figure 16 shows a real-time measurement of the frequency as a sequence of water-N_1_-water liquids circulated through the 3D-printed liquid cell. As it can be observed, the cleaning process with water, and therefore, the recovery of the initial value of f_r_, was validated with the measurement. In addition, an inset shows the frequency stability of the resonator along one minute, when the liquid cell was filled with the model solution N_1_.

**Figure 17:**
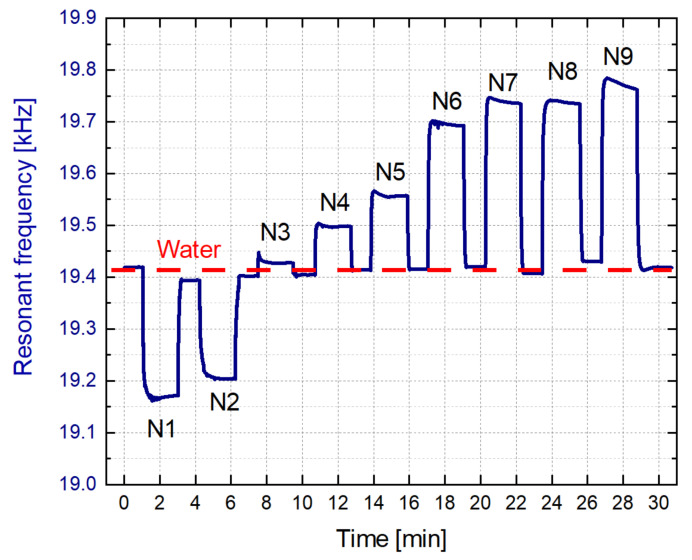
Real-time measurement of the resonance frequency while the model solutions and water circulated through the sensor.

Once the efficiency of the cleaning process was confirmed, a new test was completed. Figure 17 shows a real-time measurement of the resonant frequency as water and each model solution, from N_1_ to N_9_, continuously circulated through the liquid cell. As it is observed, the variations of the resonant frequency were obtained in real-time, detecting each one of the model solutions. In this case, the circulation of water was performed for nearly one minute and the model solutions were recirculated for two minutes. The changes in the composition of the model solutions were sensed by the resonance frequency, demonstrating the capability of our sensor

#### 3.3.2. In-Line Monitoring

**Figure 18:**
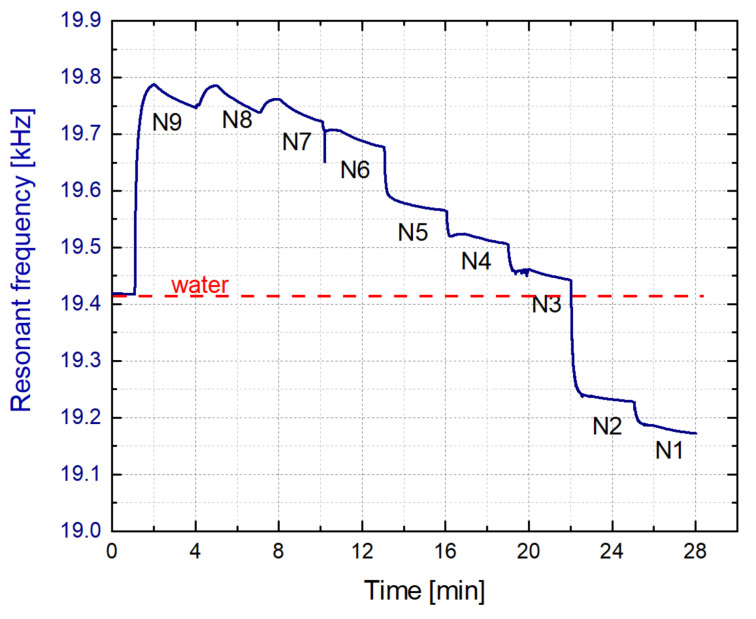
In-line monitoring of the model solutions.

In this final monitorization, named as in-line monitoring, there was a continuous flow of the model solutions of grape must, from N_9_ to N_1_, into the 3D-printed liquid cell, but without applying any cleaning process with water. As it can be observed in Figure 18, the resonance frequency was modified according to the density of each model solution in a real-time and in-line process. In this case the circulation of each model solution was about three minutes, since the frequency stabilization required a higher time compared with the previous process, where a cleaning process with water was present.

## 4. Discussion

The implementation of the 3D-printed liquid cell with embedded resonator presents as main advantages the low-cost, the simplicity and fast manufacturing, since it is straightforwardly adaptable, depending on the requirements of a final application. In addition, the PZT actuators were attached outside the 3D-liquid cell, and therefore, they were not directly immersed in the liquid, avoiding the possible deterioration of the actuators in aggressive or conductive media.

The drawback of the sensor, compared to silicon-based MEMS devices [30,31,36,37,38], could be the limited viscosity of the target liquids and the expected lower resolution. The range of viscosities that the sensor can measure depends on the quality factor of the resonances, which is lower for 3D-printed resonators as presented in this work. For example, in reference [39] with a Q-factor in isopropanol of 116, viscosities as high as 500 mPa·s were measured. To increase the Q-factor of the 3D-printed resonator, there are challenges to overcome regarding design of the cell, printing materials, different geometries, or more complex vibration modes. In addition, the long-term aging of the 3D printed materials with various chemicals flowing through the cell has only been tested for a few hours and would also determine the recommended lifetime of the low-cost sensor. A final point to mention is the effect of temperature. Our results have been obtained with constant room temperature, without any specific controller in the cell. In a real application, a closed-loop temperature controller could be implemented, with a heater and temperature sensor as in reference [64] or, alternatively, the effect of temperature on the resonant frequency should be compensated by the measurement system.

Due to these constraints, only aqueous solutions were considered for the application. However, due to the simulation and the optimization process performed, we were able to obtain a sensor with a density and viscosity resolution of 10 µg/mL and 3 µPa·s, respectively in water. Due to the novelty of this design, it was not possible to compare the resolution values obtained with similar 3D-printed sensors. Nevertheless, compared with other higher-cost systems, our resolutions were similar, or even superior. For example, in reference [37], where two cantilever resonators with different widths were used to measure the density and viscosity of glycerol and ethylene glycol solutions, a density resolution of 0.18 mg/mL and a viscosity resolution of 1 µPa·s were obtained.

Regarding the calibration procedure, since similar 3D-printed cell resonators in liquid media have not been reported, we can refer to piezoelectric resonators working in liquid media with higher cost and using silicon-based MEMS devices. For example, in reference [76] a model described that the frequency shift and motional resistance of a crystal resonator depends linearly on the square root of the viscosity-density product. This model was applied in reference [77] to a tuning fork resonator but introducing a complex impedance function determined by the fluid properties. This model, based on two calibration coefficients, was applied in several studies for the determination of the density and viscosity [78,79,80]. In reference [81] an additional parameter was introduced for measuring the density and viscosity in a gaseous environment. Finally, a modified model based in the relationship between the resonance parameters and the distributed damping associated with the liquid (g_1_) and the distributed equivalent added mass (g_2_) was established [38,69,70]. Nevertheless, the outcome of this model was only available for simple geometries and particular cases [71]. For this reason, in a previous work [82] different calibration models with different calibration coefficients, based on a Taylor series expansion of g_1_ and g_2_ in terms of the density and viscosity, were compared and solved iteratively to obtain the density and viscosity values with the lowest calibration error. In our work, a model based on four calibration coefficients was applied to obtain the density and viscosity values as presented in previous sections.

Further details of density and viscosity resolutions are presented below in Table 5, confirming the adequate performance of our sensor in comparison to other values previously reported for higher-cost systems based on MEMS devices, and therefore, demonstrating its potential as a low-cost real-time density-viscosity sensor for fluid viscosities similar to water.

Regarding the final application, this paper also contributed to the automation and the improvement of the monitoring of a grape must fermentation process. This process is traditionally monitored by enologists, in a time-consuming and high-cost activity, who manually extract and analyze discrete samples at least twice a day using an aerometer with a density resolution of 1 µg/mL [84]. Although this resolution is better than that presented here, our sensor is low-cost and portable and is capable of unattended real-time measurements throughout a fermentation process.

## 5. Conclusions

This work demonstrates a novel 3D-printed cell resonator for the real-time monitoring of the density and the viscosity of aqueous solutions, validated with the modeling of a grape must fermentation process. The device features low-cost PZT piezoelectric patches, attached to a 3D-printed cuboid-shaped liquid cell. The electrical isolation between the liquid under test and the actuators, and the fluid loading only one side of the membrane resonator, are additional advantages of the device. However, the relatively low-quality factor of the vibration mode, compared to other MEMS resonators, limits the range of densities and viscosities where the sensor can be utilized and the resolution obtained. Two important parameters of the resonator were measured using a PLL-based oscillator circuit: the quality factor and the resonant frequency. Once these two parameters were known, the viscosity and the density of the liquid could be determined. In order to measure these parameters in a real grape must, a calibration procedure of the resonator was performed using model solutions of artificial grape must, representing an ordinary fermentation process. Furthermore, an in-line flow-through monitoring of model solutions of grape must was carried out. Our results demonstrate the capability of the sensor to detect the decrease in sugar and the increase in ethanol concentrations during the grape must fermentation with a resolution of 10 µg/mL and 3 µPa·s for the density and viscosity, respectively. Regarding the sensitivity of the sensor, the results probed that the sensitivity of the resonant frequency to the density is greater with a value of 4857 Hz/g/mL.

The resolutions and sensitivity values obtained in this work confirmed the potential of our prototype as a low-cost density-viscosity sensor. Despite the limited measurement range, associated with quality factors lower than other MEMS resonators, a novel 3D-printed cell resonator for liquid media has demonstrated resolutions comparable to higher cost solutions, more difficult to use in unattended real-time processes as described in the discussion.

## Figures and Tables

**Table 1:** Key dimensions (in mm) of the PZT actuators and vibrating membrane. Wc is the width of the cell wall anchoring the membrane contour.

	PZT Actuator	Vibrating Membrane
Length	Lp = Lm/2 + Wc = 13	Lm = 16
Width	Wp = 5	Wm = 5
Thickness	Tp = 0.1	Tm = 0.5

**Table 2:** Mechanical properties of the materials used in the simulation process.

	PZT Actuator	Liquid Cell (Rigid 10K Resin)
Young Modulus [GPa]	62	10
Density [kg/m^3^]	7800	1670

**Table 4:** Composition, density and viscosity (measured with the commercial instrument) of the model solutions that represent a normal fermentation process (N_1_:N_9_).

Solution	Fructose	Glucose	Glycerol	Ethanol	Density	Viscosity
	[g/L]	[g/L]	[g/L]	[% *v*/*v*]	[g/mL]	[mPa·s]
N_1_	110	100	0	0	1.082	1.948
N_2_	90	80	0	1	1.063	1.974
N_3_	70	30	5	6	1.028	1.722
N_4_	60	20	5	8	1.017	1.706
N_5_	40	10	6	9	1.003	1.769
N_6_	20	2	7	12	0.990	1.754
N_7_	8	2	7	13	0.984	1.769
N_8_	5	2	7	13	0.983	1.808
N_9_	2	1	9	14	0.982	1.741

**Table 5:** Density and viscosity resolution compared with other works.

Liquids	Density Range	Density Resolution	Viscosity Range	Viscosity Resolution	Reference
	[g/mL]	[g/mL]	[mPa·s]	[mPa·s]	
Aqueous solutions	0.98–1.08	10 × 10^−6^	1.8–2	3 × 10^−3^	This work
Real fermentation of grape must	0.98–1.1	1 × 10^−3^	1.6–2.4	20 × 10^−3^	[29]
Solvents	0.71–0.88	0.11	2.71–44	0.43–14	[83]
Lubricant-diesel Mixtures	0.84–0.85	8 × 10^−6^–4 × 10^−5^	60–90	0.12–0.022	[64]
Viscosity standards	0.83–0.87	1.5 × 10^−7^–6.4 × 10^−4^	4–500	1.3 × 10^−4^–6.4	[39]
Glycerol solutions	0.995–1.15	0.18 × 10^−3^	0.935–4	1 × 10^−3^	[37]
